# Promoter methylation analysis of WNT/β-catenin pathway regulators and its association with expression of *DNMT1* enzyme in colorectal cancer

**DOI:** 10.1186/s12929-014-0073-3

**Published:** 2014-08-09

**Authors:** Nader Mansour Samaei, Yaghoub Yazdani, Reza Alizadeh-Navaei, Hossein Azadeh, Touraj Farazmandfar

**Affiliations:** 1 Golestan Research Center of Gastroenterology and Hepatology-GRCGH, Golestan University of Medical Sciences, Gorgan, Iran; 2 Molecular and Cell Biology Research Center, Mazandaran University of Medical Sciences, Sari, Iran; 3 Cancer Research Center, Mazandaran University of Medical Sciences, Sari, Iran

**Keywords:** WNT/β-catenin signaling, Colorectal cancer, Promoter methylation, DNMT1

## Abstract

**Background:**

Aberrant DNA methylation as the most important reason making epigenetic silencing of genes is a main mechanism of gene inactivation in patients with colorectal cancer. In this study, we decided to identify promoter methylation status of ten genes encoding WNT negative regulators, and measure the expression of *DNMT1* enzyme in colorectal cancer samples.

**Results:**

Aberrant methylation of *APC* gene was statistically significant associated with age over 50 (p = 0.017), *DDK3* with male (p < 0.0001), *SFRP4*, *WIF1*, and *WNT5a* with increasing tumor stage (p = 0.004, p = 0.029, and p = 0.004), *SFRP4* and *WIF1* with tumor differentiation (p = 0.009 and p = 0.031) and *SFRP2* and *SFRP5* with histological type (p = 0.001 and p = 0.025). The increasing number of methylated genes correlated with the expression levels of the *DNMT1* mRNA.

**Conclusions:**

The rate of gene promoter methylation of WNT pathway regulators is high in colorectal cancer cells. Hyper-methylation is associated with increased expression of the *DNMT1* enzyme.

## Background

Colorectal cancer (CRC) is the second-leading cause of cancer deaths among adults [1]. Aberrant DNA methylation as the most important reason of epigenetic gene silencing is the main mechanism of gene inactivation in patients with CRC [2],[3]. Methylation normally occurs in 5-carbon position of cytosine base within CpG dinucleotide in areas of repetitive DNA outside of exons, introduced by DNA methylases; mainly DNA methyltransferase 1 (*DNMT1*). Although the *DNMT1* enzyme is not the only factor involving in the DNA methylation, but plays a significant role in creating it [4]-[6]. DNA methylation of CpG islands in promoter regions of tumor suppressor genes and DNA repair genes is now recognized as a prevalent feature of human cancers [2].

The developmental WNT/β-catenin signaling pathway is one of the well-known signaling pathways involved in embryogenesis and cancer. A common mechanism of carcinogenesis through WNT/β-catenin signaling is the increased levels of β-catenin protein, which are often results from mutations in the adenomatous polyposis coli (*APC*) gene (encoding a β-catenin inhibitor) or in the β*-catenin* gene. Moreover, other mechanisms like functional reduction or loss of negative regulators by DNA methylation or overexpression of oncogenic ligands may contribute to WNT pathway activation in cancer development [7]-[9]. In previous studies, DNA methylation of some WNT inhibitors such as *APC*, axis inhibition protein 2 (*AXIN2*), secreted frizzled-related protein (*SFRP*) family and members of the DICKKOPF (*DKK*) have been reported in CRC [10]-[13].

As of yet, no comprehensive survey on promoter methylation levels of WNT pathway inhibitors in CRC has been performed, while a potential use of promoter methylation in cancer diagnosis and prediction can be highly regarded. In this study, promoter methylation status of ten genes encoding WNT negative regulators was assayed in CRC samples. These genes including extracellular WNT inhibitors [WNT inhibitory factor-1 (*WIF1*), *DKK3*, *SFRP2*, *SFRP4*, *SFRP5*], cytosolic WNT antagonists (*AXIN2*, *APC*), nuclear proteins [sex determining region Y related box 7 (*SOX7*) and 17 (*SOX17*)], and one WNT ligand [wingless-type MMTV integration site family member 5A (*WNT5A*)]. To estimate the association between methylation and expression of the *DNMT1* enzyme, the expression of this enzyme was measured in the samples.

## Methods

### Subjects

Samples including 125 Formalin-fixed paraffin-embedded (FFPE) cancerous and adjacent normal tissues (normal tissues distances from the tumor were not measured in this study) obtained from the CRC patients who had surgery between 2005 and 2011 from pathology laboratory. Clinical and pathologic features of patients were extracted from medical records and Tumor staging was determined on the basis of the tumor node metastasis (TNM) classification [14]. Samples with mutational familial adenomatous polyposis or hereditary non-polyposis CRC were excluded of this study. This study was approved of the Clinical Research Ethics Committee in Golestan University of Medical Science.

### Methylation analysis

Genomic DNA of microdissected tissue samples was extracted and bisulfite treated using the EpiTect Fast Bisulfite Conversion Kit (Qiagen, Hilden, Germany). Methylation specific PCR (MSP) primers of 10 selected candidate genes were prepared as described previously [15]. MSP was performed using 50 nanograms of modified DNA and Taq DNA polymerase (Roche, Woerden, Netherlands) in 7500 Real-Time PCR system (Applied Biosystem, USA). The methylation was calculated from threshold cycles (CT) values. Ten MSPs for the WNT inhibitors were analytically validated using methylated DNA as positive control (CTs ≤ 25 cycles) and primary keratinocyte DNA as unmethylated controls (CTs > 35 cycles).

### DNMT1 expression assay

The amplification primers for human *DNMT1* and Hypoxanthine-guanine phosphoribosyltransferase (*HPRT*) cDNA designed by GENE RUNNER software and be reviewed in NCBI and BLAST. *DNMT1* Primers sequence including 5′-AGCCAGGTAGCCCTCCTC-3′ as forward and 5′-GACAGCTTAACAGAAAAGGAATG-3′ as reverse with a 141 base pairs (bp) PCR product (GenBank accession number NM_001379.1) and *HPRT* including 5′-TGGACTAATTATGGACAGGACT-3′ as forward and 5′-CCTGTTGACTGGTCATTACAAT-3′ as reverse with a 219 bp PCR product (GenBank accession number NM_000194). Total RNA was purified from microdissected FFPE tissues using the PureLink FFPE RNA Isolation Kit (Invitrogen, Karlsruhe, Germany). The first strand cDNA was generated using a SuperScript III First-Strand Synthesis SuperMix (Invitrogen, Karlsruhe, Germany). Reverse transcriptase PCR was performed using SYBR Green PCR Master Mix (Fermentas, Lithuania) in triplicate and normalized to *HPRT* in 7500 detection system. Real-Time PCR conditions consist of 40 cycles (95°C for 15 s, 55°C for 30 s, 72°C for 20 s) after one step initial denaturation (95°C for 10 min). The mean and standard deviation (SD) of *DNMT1* mRNA was calculated by 2^-ΔCT^ and the expression fold was calculated by the 2^-ΔΔCT^ and a fold change ≥1.5 was considered as overexpression [16].

### Statistical analysis

Statistical analysis was performed by SPSS (version 17.0). Methylation frequencies associations with clinical and pathologic features were analyzed using chi-squared and fisher’s exact test. The survival curves were estimated by the Kaplan-Meier method. Association between promoter methylation status with survival was examined by the log-rank test. Survival time was determined from the diagnosis date to the death date due to CRC. A p-value of less than 0.05 was considered statistically significant.

## Results

### The promoter methylation status

In this study, the promoter methylation was examined on ten genes encoding the WNT pathway inhibitors in CRC tissue samples. Aberrant promoter methylation was detected in 78.4% of patients (98 of 125) (CTs ≤ 25) and none of adjacent non-tumor samples showed methylation (CTs > 35 cycles). The promoter methylation status of the ten genes, including *APC*, *AXIN2*, *DKK3*, *SFRP2*, *SFRP4*, *SERP5*, *SOX7*, *SOX17*, *WIF1* and *WNT5a* are respectively 44 samples (35.2%), 41 (32.8%), 50 (40%), 58 (46.4%), 38 (28.8%), 33 (26.4%), 40 (32%), 42 (33.6%), 52 (41.6%) and 28 (22.4%) (Table 1).

**Table 1:** **The association of promoter methylation frequencies and** ***DNMT1*** **expression with some pathological and clinical features of patients**

**Clinical and pathological features**	**N**	***APC***			***AXIN2***			***DKK3***			***SFRP2***			***SFRP4***			***SFRP5***			**SOX7**			***SOX17***			***WIF1***			***WNT5a***			***DNMT1*** **expression**		
		**N (%)**			**N (%)**			**N (%)**			**N (%)**			**N (%)**			**N (%)**			**N (%)**			**N (%)**			**N (%)**			**N (%)**			**Fold change (%)**		
		**M**	**U**	***p***	**M**	**U**	***P***	**M**	**U**	***p***	**M**	**U**	***p***	**M**	**U**	***p***	**M**	**U**	***p***	**M**	**U**	***p***	**M**	**U**	***p***	**M**	**U**	***p***	**M**	**U**	***p***	**≥1.5**	**<1.5**	***p***
**All cases**	**125**	**44 (35.2)**	**81 (64.8)**		**41 (32.8)**	**84 (67.2)**		**50 (40)**	**75 (60)**		**58 (46.4)**	**67 (53.6)**		**38 (30.4)**	**87 (69.6)**		**33 (26.4)**	**92 (73.6)**		**40 (32)**	**85 (68)**		**42 (33.6)**	**83 (66.4)**		**52 (41.6)**	**73 (58.4)**		**28 (22.4)**	**97 (77.6)**		**52 (41.6)**	**73 (58.4)**	
**Age**				.017																														
<50	**18**	11 (25)	7 (8.7)		7 (17.1)	11 (13.1)		8 (16)	10 (13.3)		9 (15.5)	9 (13.4)		5 (13.2)	13 (14.9)		8 (24.2)	10 (10.7)		7 (17.5)	11 (12.5)		10 (23.8)	8 (9.6)		5 (9.6)	13 (17.8)		6 (21.4)	12 (12.4)		7 (13.5)	11 (15.1)	
≥50	**107**	33 (75)	74 (91.3)		34 (82.9)	73 (86.9)		42 (84)	65 (86.7)		49 (84.5)	58 (86.6)		33 (86.8)	74 (85.1)		25 (75.8)	82 (89.3)		33 (82.5)	74 (87.5)		32 (76.2)	75 (90.4)		47 (90.4)	60 (82.2)		22 (78.6)	85 (87.6)		45 (86.5)	62 (84.9)	
**Sex**										<.001																								
Female	**55**	21 (47.7)	34 (42)		16 (39)	39 (46.4)		12 (24)	43 (57.3)		28 (48.3)	27 (40.3)		13 (34.2)	42 (48.3)		17 (51.5)	38 (41.3)		19 (47.5)	36 (42.4)		15 (35.7)	40 (48.2)		23 (44.2)	32 (43.8)		12 (42.8)	43 (44.3)		21 (40.4)	34 (46.6)	
Male	**70**	23 (52.3)	47 (58)		25 (61)	45 (53.6)		38 (76)	32 (42.7)		30 (51.7)	40 (59.7)		25 (65.8)	45 (51.7)		16 (48.5)	54 (58.7)		21 (52.5)	49 (57.6)		27 (64.3)	43 (51.8)		29 (55.8)	41 (56.2)		16 (57.2)	54 (55.7)		31 (59.6)	39 (53.4)	
**Tumor location**																																		
Proximal	**58**	18 (40.9)	40 (49.2)		19 (46.3)	39 (46.4)		24 (48)	34 (45.3)		32 (55.1)	26 (38.8)		17 (44.7)	41 (47.1)		16 (48.5)	42 (45.7)		21 (52.5)	37 (43.5)		20 (47.6)	38 (45.8)		27 (51.9)	31 (42.5)		14 (50)	44 (45.4)		28 (53.8)	30 (41.1)	
Distal	**67**	26 (59.1)	41 (50.8)		22 (53.7)	45 (53.6)		26 (52)	41 (54.7)		26 (44.9)	41 (61.2)		21 (55.3)	46 (52.9)		17 (51.5)	50 (54.3)		19 (47.5)	48 (56.5)		22 (52.4)	45 (54.2)		25 (48.1)	42 (57.5)		14 (50)	53 (54.6)		24 (46.2)	43 (58.9)	
**TNM stage**																.004												.029			.004			
I	**17**	5 (11.4)	12 (14.8)		6 (14.6)	11 (13.1)		8 (16)	9 (12)		9 (15.5)	8 (11.9)		4 (10.5)	13 (14.9)		5 (15.2)	12 (13)		7 (17.5)	10 (11.8)		6 (14.3)	11 (13.3)		12 (23.1)	5 (6.8)		3 (10.7)	14 (14.4)		7 (13.5)	10 (13.7)	
II	**39**	17 (38.6)	22 (27.2)		11 (26.8)	28 (33.3)		15 (30)	24 (32)		18 (31)	21 (31.3)		10 (26.3)	29 (33.3)		12 (36.4)	27 (29.3)		13 (32.5)	26 (30.6)		10 (23.8)	29 (34.9)		14 (26.9)	25 (34.3)		5 (17.8)	34 (35.1)		13 (25)	26 (35.6)	
III	**61**	18 (40.9)	43 (53.1)		19 (46.4)	42 (50)		22 (44)	39 (52)		25 (43.1)	36 (53.8)		17 (44.7)	44 (50.6)		13 (39.4)	48 (52.2)		16 (40)	45 (52.9)		24 (57.1)	37 (44.6)		25 (48.1)	36 (49.3)		14 (50)	47 (48.4)		27 (51.9)	34 (46.6)	
IV	**8**	4 (9.1)	4 (4.9)		5 (12.2)	3 (3.6)		5 (10)	3 (4)		6 (10.4)	2 (3)		7 (18.5)	1 (1.2)		3 (9)	5 (5.5)		4 (10)	4 (4.7)		2 (4.8)	6 (7.2)		1 (1.9)	7 (9.6)		6 (21.5)	2 (2.1)		5 (9.6)	3 (4.1)	
**Tumor differentiation**																.009												.031						
Low	**11**	4 (9.1)	7 (8.7)		5 (12.2)	6 (7.1)		3 (6)	8 (10.7)		4 (6.9)	7 (10.4)		6 (15.8)	5 (5.7)		3 (9.1)	8 (8.7)		2 (5)	9 (10.6)		7 (16.7)	4 (4.8)		1 (1.9)	10 (13.7)		3 (10.7)	8 (8.2)		4 (7.7)	7 (9.6)	
Moderate	**89**	30 (68.2)	59 (72.8)		27 (65.9)	62 (73.8)		39 (78)	50 (66.7)		43 (74.1)	46 (68.7)		20 (52.6)	69 (79.3)		22 (66.7)	67 (72.8)		32 (80)	57 (67.1)		30 (71.4)	59 (71.1)		37 (71.2)	52 (71.2)		18 (64.3)	71 (73.2)		36 (69.2)	53 (72.6)	
High	**25**	10 (22.7)	15 (18.5)		9 (21.9)	16 (19.1)		8 (16)	17 (22.6)		11 (19)	14 (20.9)		12 (31.6)	13 (15)		8 (24.2)	17 (18.5)		6 (15)	19 (22.3)		5 (11.9)	20 (24.1)		14 (26.9)	11 (15.1)		7 (25)	18 (18.6)		12 (23.1)	13 (17.8)	
**Histological type**													.001						.025															
Non-mucinous	**98**	30 (68.2)	68 (84)		34 (82.9)	64 (76.2)		39 (78)	59 (78.7)		53 (91.4)	45 (67.2)		30 (78.9)	68 (78.2)		21 (63.6)	77 (83.7)		34 (85)	64 (75.3)		32 (76.1)	66 (79.5)		37 (71.2)	61 (83.6)		19 (67.8)	79 (81.4)		35 (67.3)	63 (86.3)	
Mucinous	**27**	14 (31.8)	13 (16)		7 (17.1)	20 (23.8)		11 (22)	16 (21.3)		5 (8.6)	22 (32.8)		8 (21.1)	19 (21.8)		12 (36.4)	15 (16.3)		6 (15)	21 (24.7)		10 (23.9)	17 (20.5)		15 (28.8)	12 (16.4)		9 (32.2)	18 (18.6)		17 (32.7)	10 (13.7)	

N; Number of cases, M; Methylated, U; Unmethylated, *p*; *p* value.

Only significant *P* values are described.

### The correlation with clinical and pathological features

We analyzed methylation status relationship of these genes with some patients’ clinical and pathological features. As the results in Table 1 show, *APC* gene methylation is statistically associated with age over 50 (p = 0.017). *DDK3* gene methylation is also significantly associated with male (p < 0.0001). Methylation of *SFRP4*, *WIF1* and *WNT5a* genes were meaningfully associated with increasing tumor stage (p = 0.004, p = 0.029 and p = 0.004). Moreover, methylation frequency of *SFRP4* and *WIF1* genes were also significantly associated with tumor differentiation (p = 0.009 and p = 0.031). The *SFRP2* and *SFRP5* genes methylation was correlated with histological type, therefore the frequency of methylation is higher in non-mucinous type (p = 0.001 and p = 0.025). There is no significant association between genes methylation status and tumor location. There is also no significant association between *DNMT1* expression and clinicopathological features.

**Figure 1:**
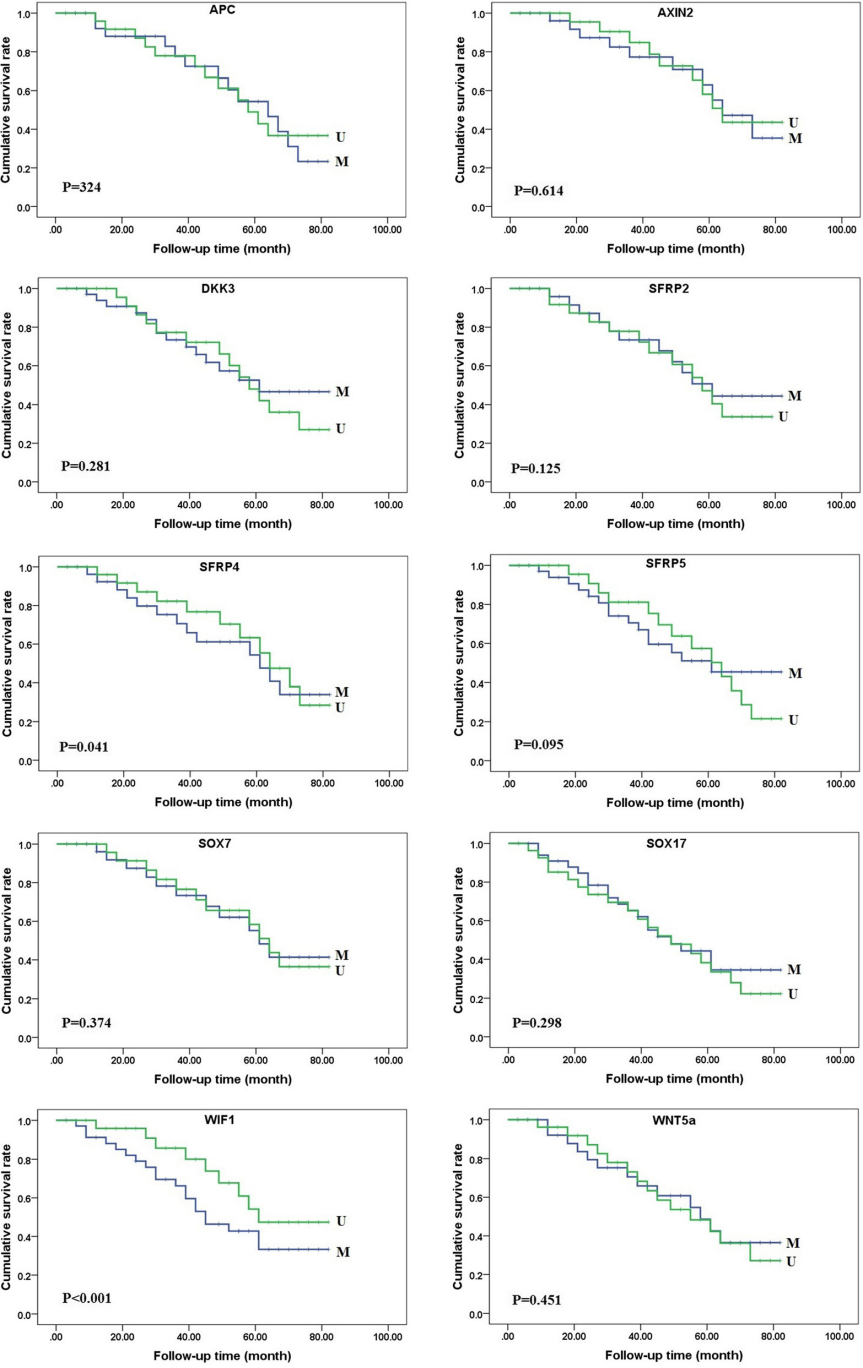
**Kaplan-Meier curves for overall survival in colorectal carcinomas.** Kaplan-Meier curves for cumulative survival rate of patients according to the promoter methylation in all the colorectal carcinomas. Among the ten genes, only patients with methylated *WIF1* gene promoter had shorter overall survival. M, Methylated; U,Unmethylated.

Follow-up information was available on 112 CRC patients for 42 ± 25 (3 to 80) months. Univariate analysis by the Kaplan-Meier curves indicated, among the ten genes, only *WIF1* has a negative correlation between promoter methylation and survival in CRC patients (P < 0.001) (Figure 1).

### Correlation of *DNMT1* overexpression with the increasing number of methylated genes

**Figure 2:**
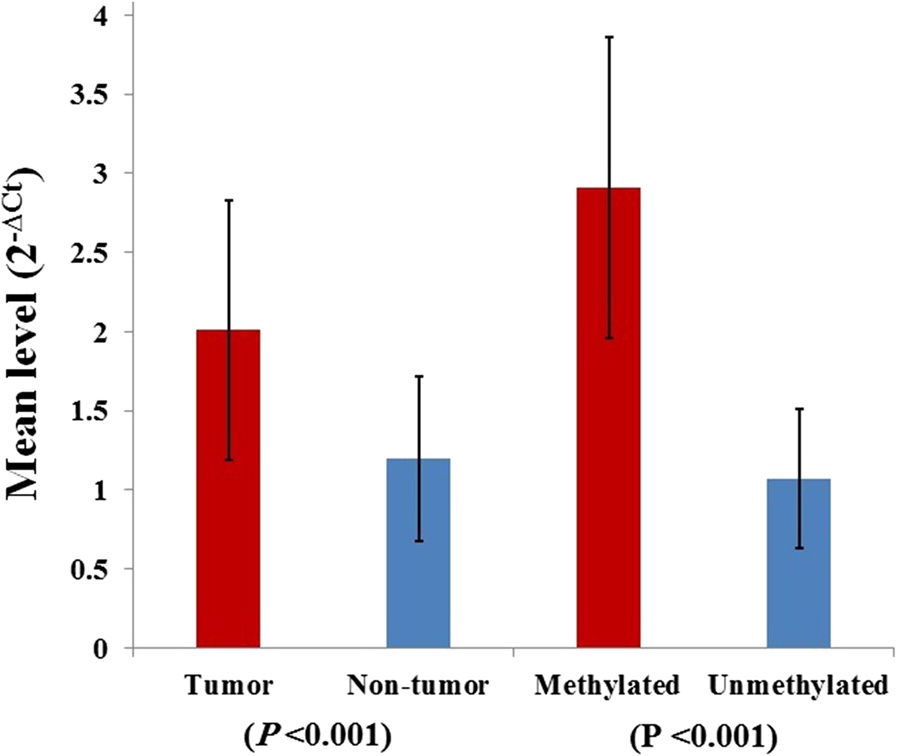
**The expression levels of*DNMT1*enzyme mRNA in colorectal carcinomas.** The level of *DNMT1* expression in methylated samples is higher than non-methylated as well as tumor samples than non-tumor.

We assayed the expression levels of *DNMT1* enzyme mRNA in 125 tumor and adjacent normal tissues. As Figure 2 shows, *DNMT1* expression in CRC tissues (2.01 ± 0.82) is statistically higher than the non-tumor mucosa (1.02 ± 0.52) (P < 0.001), as well as methylated (2.91 ± 0.95) than non-methylated samples (1.07 ± 0.44) (P < 0.001). We also investigated the expression levels of *DNMT1* mRNA within methylated samples and was compared to fold changes of *DNMT1* expression. For this purpose, methylated samples were divided into six groups according to the number of methylated genes. The results show; the expression level of the *DNMT1* mRNA was directly correlated with the increasing number of methylated genes in methylated samples (Table 2).

**Table 2:** **The relationship of** ***DNMT1*** **expression with the number of methylated genes**

**Number of methylated genes**	***DNMT1*-overexpressed cases**		**Odds ratio (CI 95%)**
	**N (%)**	**Fold change (mean ± SD)**	
1 (n = 35)	13 (37.1)	1.52 ± 1.12	1
2 (n = 19)	11 (57.9)	2.12 ± 1.56	2.3 (0.7-7.2)
3 (n = 16)	9 (56.2)	2.02 ± 1.78	2.1 (0.6-7.2)
4 (n = 10)	6 (60)	3.61 ± 1.89	2.5 (0.6-10.7)
5 (n = 11)	7 (63.6)	3.06 ± 1.55	2.9 (0.7-12.9)
6 (n = 7)	6 (85.7)	4.14 ± 1.28	10.1 (1.1-93.9)*

*p value <0.05, CI, confidence interval.

## Discussion

Today, the epigenetic markers of causing changes in the WNT signaling pathways are highly considered, both in the detection and prognosis of the disease process in many cancers [17]. The study of methylation as a reversible major change during the early stages of tumor angiogenesis is under investigation [18],[19]. Hence, we were going to investigate the variability of promoter methylation of WNT negative regulators to estimate these changes in CRC. In this study, the frequency of promoter methylation of *APC* gene (35.2%) is higher than the frequency obtained during the studies of Chen et al. (17.9%), Esteller et al. (18%) and Arnold et al. (28%) [10],[20],[21]. On the other hand, this frequency is less than the frequencies in Studies of Iacopetta et al. (41.1%) and Fu et al. (41.7%) [22],[23]. Because, changes in the *APC* gene are the most common cause of CRC, 35.2% methylation can also be significant. The *AXIN2* methylation frequency of this study (32.8%) compared to prevalence of the Koinuma et al. study (25%) had higher level [11]. It seems that frequency of *DKK3* promoter methylation (40%) is less than Yu et al. study (52%). Unlike Yu et al. study, we reported that the increasing of promoter methylation frequency was significant in men, but we did not find a significant association between promoter methylation frequencies with cancer progression stages [12]. The promoter methylation frequency of *SFRP2, 4, 5* genes in this study (46.4%, 28.8%, and 26.4%) compared to the results of Qi et al. study (82.8%, 32.4% and 54.3%) is much lower [13]. Low frequency of *WIF1* promoter methylation in this study (41.6%) compared to the Lee et al. study (74%) was also considerable [24]. There are few differences between frequency of *WNT5a* promoter methylation in this study (22.4%) and Rawson et al. study (18.8%). In both studies, the frequency of promoter methylation is significantly associated with female gender [25] (Table 3). Univariate analysis indicates that WIF1 promoter methylation is prognostic factors for overall survival in colorectal carcinomas. The results of this study show that *DNMT1* expression levels in methylated samples are higher from unmethylated, unlike Ting et al. study in which they believed the DNMT1 enzyme has no effect on the maintenance of DNA methylation [26]. The increasing expression of the DNMT1 enzyme weakly correlates with the increasing number of methylated genes (Table 2). On the other hand, there have not been many studies on correlation between DNMT1 expression and methylation, which makes it difficult to evaluate our work; Therefore, It needs more study.

**Table 3:** The comparison of promoter methylation of this study and other reports in colorectal carcinomas

**Genes**	**Number of methylated samples (%)**	**References (frequency of gene)**
APC	44 (35.2)	Esteller et al. [21] (18%); Arnold et al. [10] (28%); Chen et al. [20] (17.9%); Iacopetta et al. [22] (41.1%) and Fu et al. [23] (41.7%)
AXIN2	41 (32.8)	Koinuma et al. [11] (25%)
DKK3	50 (40)	Yu et al. [12] (52%)
SFRP2	58 (46.4)	Qi et al. [13] (82.8%)
SFRP4	38 (30.4)	Qi et al. [13] (32.4%)
SFRP5	33 (26.4)	Qi et al. [13] (54.3%)
SOX7	40 (32)	No Reference
SOX17	42 (33.6)	No Reference
WIF1	52 (41.6)	Lee et al. [24] (74%)
WNT5a	28 (22.4)	Rawson et al. [25] (18.8%)

## Conclusions

We have shown that the rate of gene methylation in cancer cells increases can contribute to cancer development and progression. Methylation may also increase due to increased expression or mutations in the methyltransferase enzyme or their regulatory proteins in cancer cells. Knowing the rate of *DNMT1* enzyme overexpression in human cancers, particularly in cases of hyper-methylated might be useful as part of the diagnostic and prognostic evaluation in human cancers.

## Competing interests

The authors declare no conflict of interests.

## Authors’ contributions

NMS carried out the Real-Time PCR, data analysis and manuscript preparation. YY, RAN and HA participated project design, data analysis and manuscript preparation. TF supervised experimental design, data analysis and reviewed manuscript. All authors read and approved the final manuscript.
